# Adiponectin and resistin in acute and chronic graft-vs-host disease patients undergoing allogeneic hematopoietic stem cell transplantation

**DOI:** 10.3325/cmj.2016.57.255

**Published:** 2016-06

**Authors:** Oliver Robak, Zoya Kuzmina, Andreas Winkler, Peter Kalhs, Werner Rabitsch, Hildegard Greinix

**Affiliations:** 1 Department of Medicine I, Medical University of Vienna, Vienna, Austria; 2 Division of Hematology, Medical University of Graz, Graz, Austria

## Abstract

**Aim:**

To investigate the association of adiponectin and resistin levels in patients undergoing hematopoietic stem cell transplantation (HSCT) with the clinical outcome, including the occurrence of acute and chronic graft-vs-host disease (GVHD), non-relapse mortality, and overall survival.

**Methods:**

We prospectively collected serum samples from 40 patients undergoing either autologous (n = 12; 10 male) or allogeneic (n = 28; 11 male) HSCT for up to 12 months post HSCT and determined adiponectin and resistin serum concentrations using enzyme-linked immunosorbent assay.

**Results:**

There were no significant differences in adiponectin levels (18.5 vs 9.3 µg/mL, *P* = 0.071) and adiponectin/BMI ratio (0.82 vs 0.39, *P* = 0.068) between patients with acute GVHD grades 2-4 and autologous controls. However, resistin values were significantly lower in patients with acute GVHD grades 2-4 than in autologous controls (4.6 vs 7.3 ng/mL, *P* = 0.030). Adiponectin levels were higher in patients with chronic GVHD (n = 17) than in autologous controls (13.5 vs 7.6 µg/mL, *P* = 0.051), but the difference was not significant. Adiponectin/BMI ratio was significantly higher in patients with chronic GVHD than in autologous controls (0.59 vs 0.25, *P* = 0.006). Patients dying from relapse also had significantly lower adiponectin levels (8.2 µg/mL) and adiponectin/BMI ratio (0.3) on admission than surviving allogeneic (15.8 µg/mL, *P* = 0.030 and 0.7, *P* = 0.004) and surviving autologous patients (19.2 µg/mL, *P* = 0.031 and 0.7, *P* = 0.021).

**Conclusion:**

Adiponectin and resistin levels were altered in patients with acute and chronic GVHD compared to autologous controls and were associated with overall survival and relapse mortality in patients undergoing allogeneic HSCT.

Allogeneic hematopoietic stem cell transplantation (HSCT) offers potential cure to an increasing number of patients with hematological diseases (1,2). However, it is still associated with substantial morbidity related in part to graft-vs-host disease (GVHD). GVHD occurs in around 50% of recipients and presents a major complication (3-5). Severe GVHD is associated with reduced survival (6) and impaired quality of life (7). Chronic GVHD (cGVHD) is one of the main causes of non-relapse mortality (NRM) and prolonged immunodeficiency (3,4). Clinical signs of cGVHD can resemble those of autoimmune disorders such as systemic lupus erythematosus, Sjogren’s syndrome, scleroderma, autoimmune thyroiditis, and rheumatoid arthritis (8-10). In 2005, the National Institutes of Health (NIH) defined the criteria for diagnosis and severity scoring of cGVHD (11,12).

Acute GVHD (aGVHD) is an inflammatory disorder that occurs when transplanted donor T-lymphocytes react to host cells and tissues that are recognized as foreign (13). Pro-inflammatory cytokines like interleukin-1 (IL1), interleukin-6 (IL6), and tumor necrosis-factor (TNF)-alpha are up-regulated and contribute to the high morbidity and mortality. Importantly, macrophages exhibit potent regulatory functions *in vivo* with the help of T-cells (14).

Adipokines are cytokines secreted predominately by the adipose tissue. They exert a variety of distinct metabolic, endocrine, and immune functions, both locally and systemically. Adiponectin is an adipocyte-derived secretory protein, which is an important regulator of inflammatory responses (15,16). In many inflammatory states, adiponectin levels are inversely correlated with pro-inflammatory markers (17-19). Overall, adiponectin exerts predominantly anti-inflammatory effects and suppresses the proliferation of myelomonocytic progenitor cells (20,21). Furthermore, it inhibits the classical pro-inflammatory function of macrophages, promoting an M2 macrophage phenotype (22), and diminishing phagocytosis and cytokine production upon lipopolysaccharide-stimulation by interfering with nuclear factor kappa-B activation (23). This is also an important mechanism during HSCT, as recipient macrophages contribute to GVHD by antigen-presentation and secretion of cytokines, causing the activation and proliferation of CD8^+^ T cells (24,25). Moreover, adiponectin reduces T-lymphocyte recruitment via reduction of interferon-beta production (26). In a model of murine cardiac transplantation, adiponectin attenuated allograft rejection in major histocompatibility complex class II mismatched transplants (27).

Another adipokine, resistin, forms an important link between obesity, insulin resistance, and diabetes (28,29). In humans, increased levels of resistin have been found in mononuclear leukocytes and macrophages (30). Resistin has further been associated with inflammation in systemic autoimmune diseases (31) and might counteract adiponectin action with regards to macrophage function by promoting a pro-inflammatory state (32,33).

An association between serum high-molecular-weight (HMW) adiponectin levels and cGVHD severity in allogeneic HSCT recipients was ﬁrst suggested in a retrospective analysis by Nakasone et al (34). However, they investigated HMW-adiponectin only and did not take into account that adiponectin levels inversely correlated with the body mass index (BMI) (35,36). We performed a prospective study to investigate the association of total adiponectin and classical inflammatory markers and the transplant outcome including the occurrence of aGVHD and cGVHD, as well as relapse and survival. Besides adiponectin levels, we calculated the ratio of the absolute adiponectin plasma levels and BMI in order to compensate for the fact that adiponectin levels are closely correlated with adipose tissue mass and body mass index (37,38). Whereas Nakasone et al (34) compared autologous transplant recipients to healthy controls (34), we compared them to allogeneic ones, in order to have patient groups with comparable toxicity profiles related to the administration of conditioning therapies.

## Patients and methods

### Patients and study design

Between November 2008 and December 2010, we prospectively collected serum samples from patients undergoing either autologous (n = 12; 10 male) or allogeneic (n = 28; 11 male) HSCT (Table 1). Blood samples were obtained at the following time points: on admission (7 days before HSCT; T-1), on the day of HSCT (T0), during aplasia (defined by absolute neutrophil count <0.5 G/L; T+1), on the day of engraftment (defined by absolute neutrophil count >0.5 G/L; T+2), 1 month after HSCT (T+3), 3 to 6 months after HSCT (T+4), and 6 to 12 months after HSCT (T+5).

**Table 1:** Patients’ characteristics

	All N (%)	Allogeneic N (%)	Autologous N (%)
Number of patients	40 (100)	28 (70)	12 (30)
Median age in years (range)	46 (34-56)	44 (34-56)	48 (42-49)
Sex			
male	21 (53)	11 (39)	10 (83)
female	19 (47)	17 (61)	2 (17)
BMI on admission		22.9	25.8
BMI overall mean (range)	25 (17.6-33)	24.6 (17.6-33)^†^	25.8 (19-31.2)^†^
Diagnosis			
acute myeloid leukemia	21 (53)	21 (75)	0 (0)
chronic myeloid leukemia	1 (3)	1 (4)	0 (0)
lymphoma	9 (23)	5 (18)	4 (33)
myeloma	6 (15)	0 (0)	6 (50)
other^‡^	3 (8)	1 (4)	2 (17)
Disease status at transplantation			
standard risk^§^	21 (53)	15 (54)	6 (50)
high risk^§^	19 (48)	13 (46)	6 (50)
Conditioning			
myeloablative	26 (65)	14 (50)	12 (100)
Reduced-intensity conditioning	14 (35)	14 (50)	0 (0)
Stem cell donors			
related	11 (39)	11 (39)	N/A
unrelated	17 (61)	17 (61)	N/A
HLA-identical	21 (75)	21 (75)	N/A
HLA-mismatched	7 (25)	7 (25)	N/A
Stem cell source			
bone marrow	1 (3)	1 (4)	0 (0)
peripheral blood stem cells	39 (98)	27 (96)	12 (100)
Post-transplant immunosuppressive prophylaxis			
cyclosporine only	4 (10)	4 (14)	N/A
cyclosporine-methotrexat	14 (35)	14 (50)	N/A
cyclosporine-mycophenolate mofetil	10 (25)	10 (36)	N/A
Median follow-up, months (range)	26 (0.1-46)	24 (0.1-46)	30.3 (4.3-46)

*N – number of patients; BMI – body mass index; HLA – human leukocyte antigen.

†Difference between groups is statistically significant.

‡Other diagnoses included myelodysplastic syndrome and chronic lymphocytic leukemia. §Standard risk was defined as acute leukemia in the first or second complete remission or chronic myeloid leukemia in the first chronic phase. High-risk disease included myelodysplastic syndrome, acute and chronic leukemia beyond second complete remission or in relapse, as well as chronic myeloid leukemia beyond the first chronic phase.

41 consecutive patients were included into the study. 1 patient died two days after enrolment and was excluded from the analysis, leaving 40 patients for the analysis. Patients were enrolled prior to the start of myeloablative (n = 26) or reduced-intensity conditioning (RIC, n = 14) for HSCT. Autologous HSCT patients (n = 12) served as controls. The diagnosis and the severity of aGVHD and cGVHD were determined based on the modified Glucksberg and NIH classification (11,39,40). All patients received anti-infective prophylaxis as previously described (41). This study was approved by the Institutional Review Board of the Medical University of Vienna. All patients gave written informed consent in accordance with the Declaration of Helsinki.

### Enzyme-linked immunosorbent assay

Serum adiponectin and resistin concentrations were measured using enzyme-linked immunosorbent assay (ELISA) according to the manufacturer’s instructions (Resistin and Adiponectin Human ELISA, BioVendor R&D, Brno, Czech Republic). Since adiponectin levels closely correlate with adipose tissue mass and BMI (37,38), values were additionally calculated as ratio adiponectin/BMI (42). The reference range of adiponectin plasma levels is between 2 and 15 µg/mL (43,44) and that of resistin plasma levels is between 10 and 30 ng/mL (45). Serum amyloid-A (SAA), haptoglobin (HPT), and fibronectin (FNC) were measured as classical markers of inflammation by nephelometry (Behring Nephelometer, Siemens Healthcare Diagnostics GmbH, Munich, Germany).

### Statistical analysis

NRM was defined as any death not related to the underlying malignancy. Relapse was defined as recurrence of malignancy after achievement of complete remission, with NRM as a competing risk. Cumulative incidences of acute and chronic GVHD were estimated considering relapse/progression and death as a competing event. OS was calculated from the day 0 of HSCT to the day of death from any cause or last follow-up.

Normality was tested using the Kolmogorov-Smirnov test. Serum levels in patient groups were compared using unpaired *t* test in case of variables with normal distribution, otherwise the Mann-Whitney U test was used. Fisher exact test was used to test the significance of the association between two variables. Multiple linear regression was used to determine the relationship between two or more explanatory variables and a response variable. Differences were considered statistically significant at a two-sided *P* value <0.05. *P*-values were corrected for multiple testing. The data are presented as mean and standard deviation or median and interquartile range. Statistical analysis was performed using SPSS 20.0 (IBM Corp, Armonk, NY, USA).

## Results

### Demographics

The study included 40 patients. Apart from sex and disease, there were no significant differences in patient characteristics between the autologous and the allogeneic HSCT patients (Table 1). Women had slightly higher adiponectin levels and adiponectin/BMI ratios, but the difference was not significant (*P* = 0.053, *t* test) (34-36).

### Adiponectin and resistin during HSCT

Patients who received myeloablative conditioning (n = 14) had significantly lower adiponectin levels at T0 (11 vs 25.3 µg/mL, *P* = 0.028, *t* test) and significantly higher resistin levels at T+2 (2.9 vs 3.8 ng/mL, *P* = 0.029, *t* test) than patients receiving RIC (n = 14).

No other patient characteristic or clinical parameter (age, sex, BMI, donor source, HLA identity, stem cell source, and GVHD prophylaxis) had a significant impact on adiponectin and resistin levels (multiple linear regression).

### Acute GVHD

**Figure 1:**
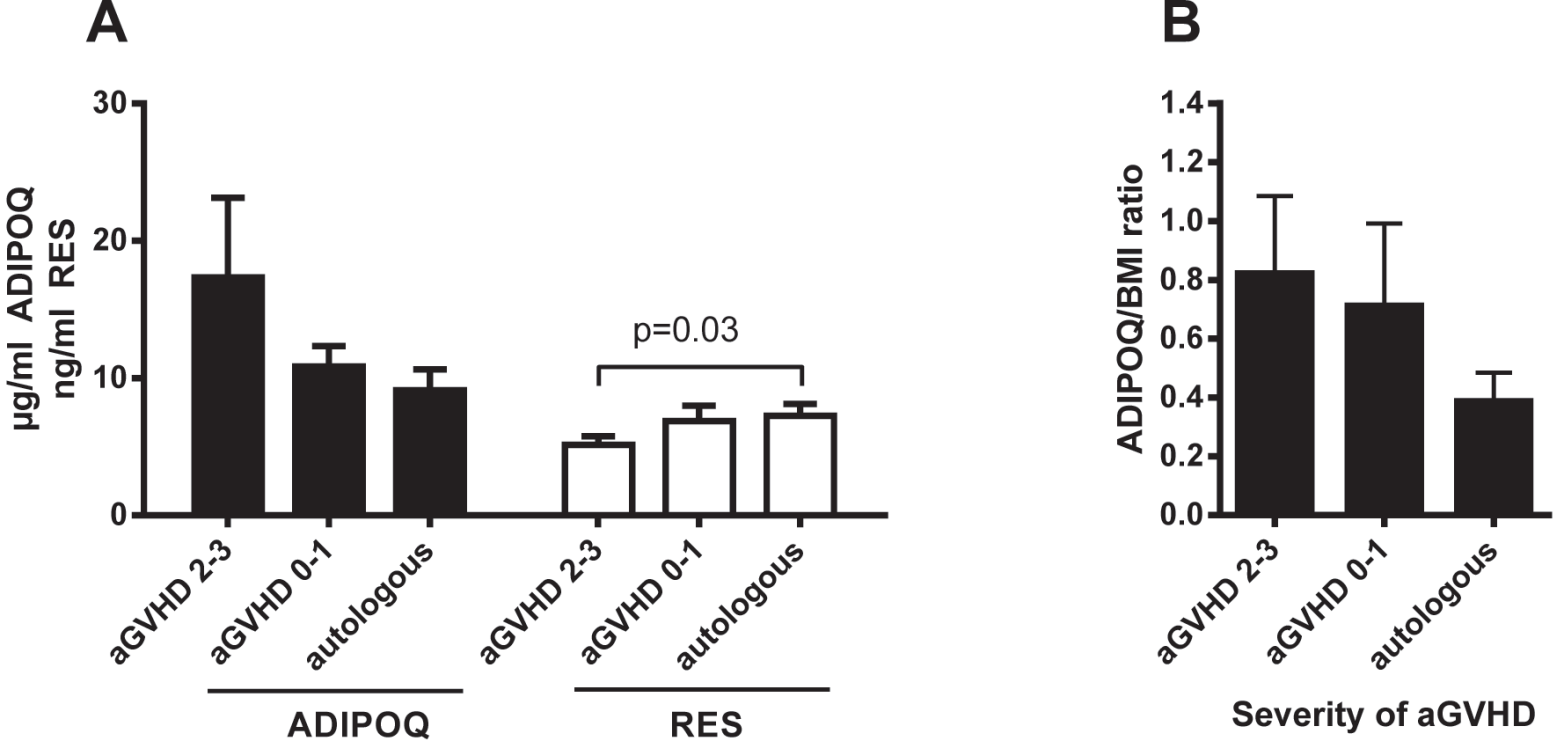
Adiponectin (ADIPOQ) and resistin (RES) levels (**A**) and ADIPOQ/body mass index (BMI) ratio (**B**) in patients with established acute acute graft-vs-host disease (aGVHD) compared to autologous controls; measured at disease maximum.

Cumulative incidence of aGVHD was 64% (n = 18) at a mean of 21.5 (16.5-32) days after HSCT, including 67% of patients (n = 12) with grades 2-4 (Table 2). Mean adiponectin levels in patients with established aGVHD grades 2-4 were 18.5 ± 9.7 µg/mL compared to 9.3 ± 4.8 µg/mL (*P* = 0.071, *t* test) in autologous HSCT controls (Figure 1A, measured at disease maximum); mean adiponectin/BMI ratio in patients with established aGVHD grades 2-4 was 0.82 ± 0.3 compared to 0.39 ± 0.30 (*P* = 0.068, *t* test) in autologous controls (Figure 1B, measured at disease maximum). Mean resistin levels in patients with established aGVHD were 4.6 ± 3.3 ng/mL compared to 7.3 ± 2.1 ng/mL in autologous controls (*P* = 0.030, *t* test, Figure 1A, measured at disease maximum). Resolution of aGVHD without later development of cGVHD (n = 7) was associated with a decrease in adiponectin levels to a mean of 13.2 ± 10.4 µg/mL (*P* = 0.037, Mann-Whitney-U test) and an increase in resistin levels to 13.8 ± 2.5 ng/mL (*P* = 0.033, Mann-Whitney-U test).

**Table 2:** Characteristics of acute graft-vs-host disease (aGVHD) and chronic GVHD (cGVHD)^*†^

	aGVHD N (%)	cGVHD N (%)
Total	18 (64)	17 (61)
Sex patient (female)	10 (56)	11 (65)
Sex donor (female)	8 (44)	6 (35)
CMV patient positive	11 (61)	8 (47)
CMV donor positive	12 (67)	10 (59)
Organ involvement		
skin	14 (78)	11 (65)
eyes		11 (65)
oral mucosa		8 (47)
liver	10 (56)	10 (59)
lungs		4 (24)
gastrointestinal system	10 (56)	2 (12)
joints		1 (6)
Severity score (disease maximum)	Glucksberg	NIH
1	6 (33)	10 (59)
2	7 (39)	4 (24)
3	4 (22)	3 (18)
4	1 (6)	0 (0)
Onset type of cGVHD		
de novo		6 (35)
quiescent		6 (35)
progressive		5 (29)
Median time to first onset of GVHD in days, (range)	27 (10-80)	123 (75-222)

*CMV – cytomegalovirus.

†Data displayed as cumulative incidences.

**Figure 2:**
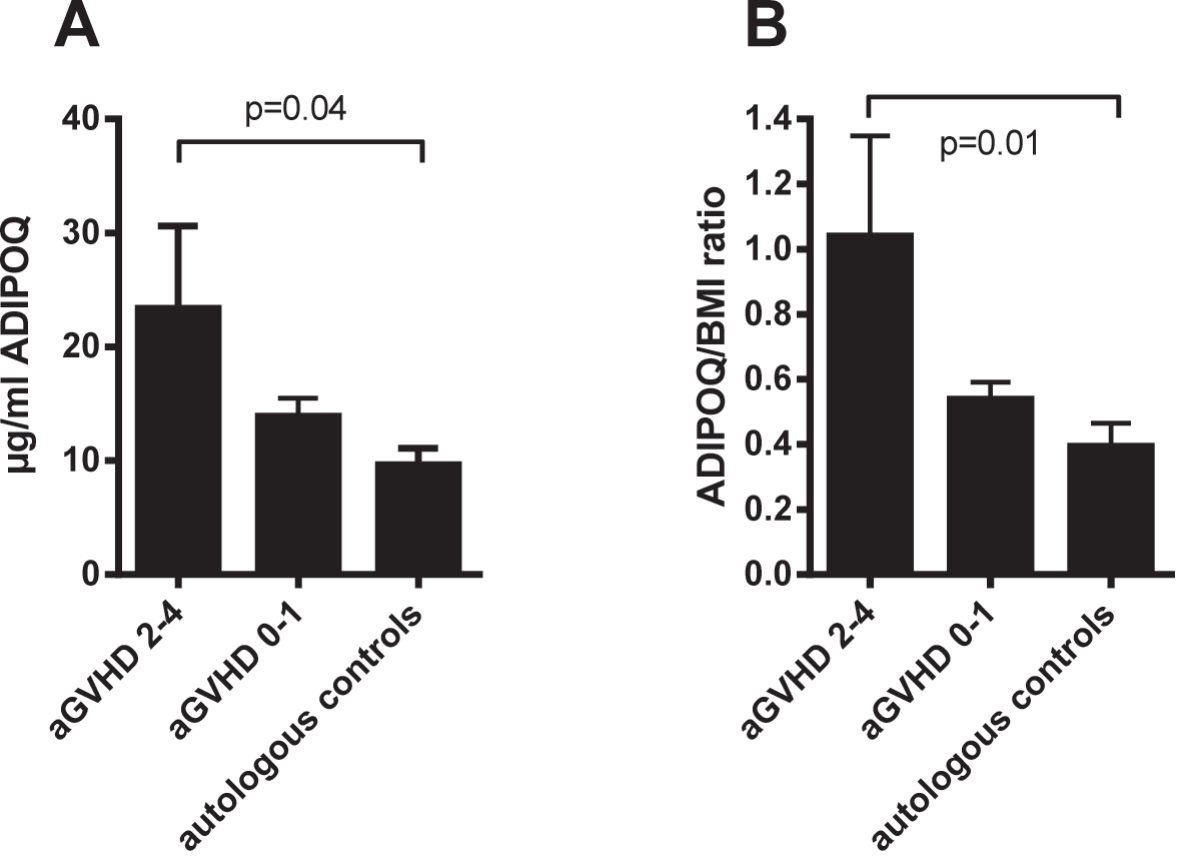
Adiponectin (ADIPOQ) (**A**) and ADIPOQ/body mass index (BMI) ratio (**B**) in patients subsequently developing acute graft-vs-host disease (aGVHD) compared to autologous controls during aplasia.

Patients with subsequent aGVHD grades 2-4 (n = 18) had significantly higher adiponectin levels (23.4 vs 9.6 µg/mL, *P* = 0.041, *t* test) and adiponectin/BMI ratios (1.0 vs 0.4, *P* = 0.008, *t* test) during aplasia than autologous controls (Figure 2). They also had significantly reduced resistin until day 32 (the day of the latest aGVHD diagnosis) than controls at T+1 (5.3 vs 13.3 ng/mL, *P* = 0.001, and 5.1 vs 7.3 ng/mL, *P* = 0.033, *t* test).

Besides a significant reduction in SAA (30.7 vs 145 µg/mL, *P* = 0.04) at engraftment and 1 month after HSCT, no differences in other classical inflammation markers were observed between patients with aGVHD grades 2-4 and autologous controls.

Except for donor source (related vs unrelated donor, 39% vs 61%, *P* = 0.050, *t* test) and BMI, no other patient characteristic or clinical parameter (age, sex, HLA identity, stem cell source, conditioning regime, and GVHD prophylaxis) had an impact on the incidence of acute GVHD (multiple linear regression).

### Chronic GVHD

**Figure 3:**
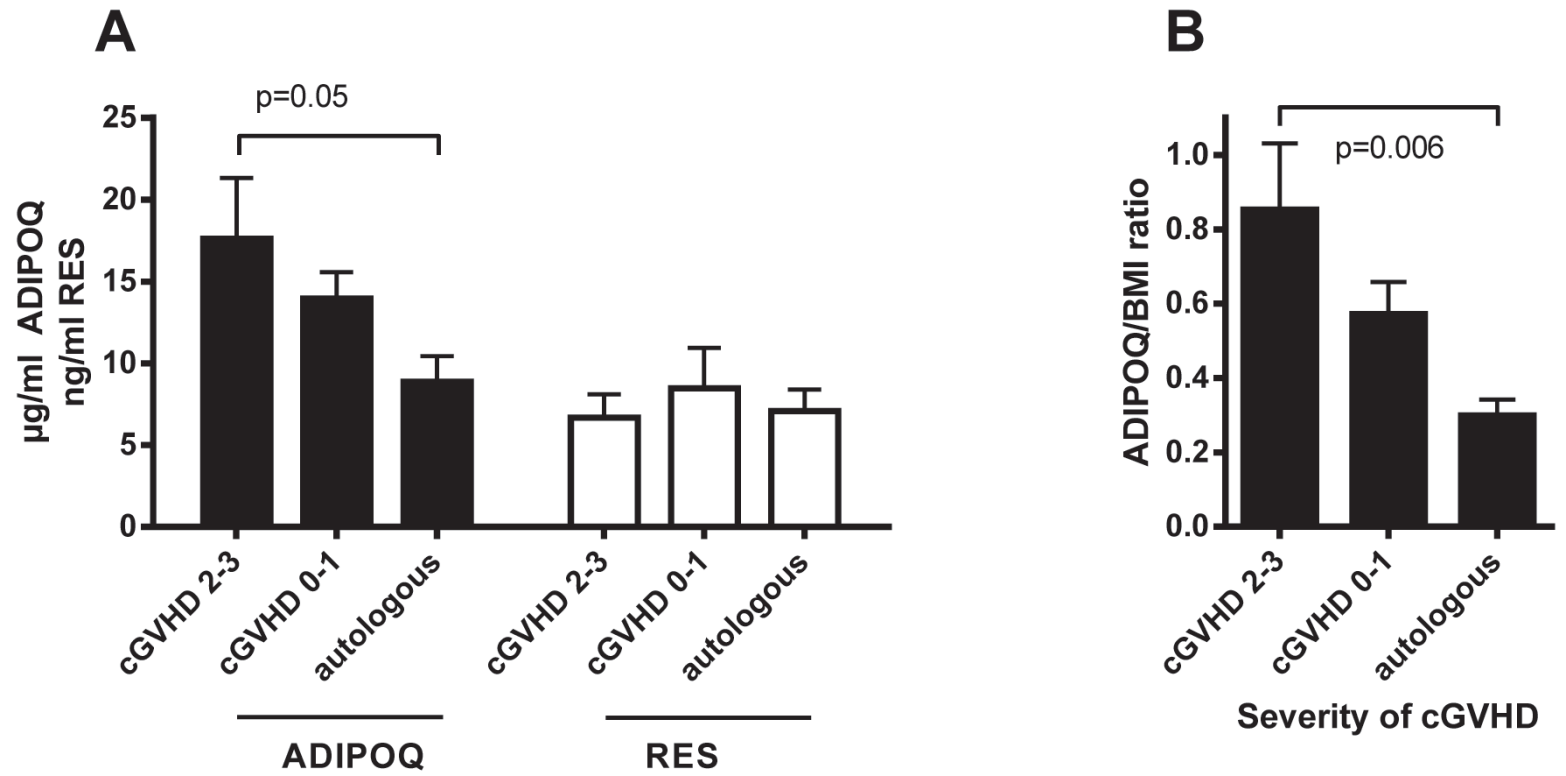
Adiponectin (ADIPOQ) and resistin (RES) levels (**A**), and ADIPOQ/body mass index (BMI) ratio (**B**) in chronic graft-vs-host disease (cGVHD) patients compared to autologous controls; measured at disease maximum.

Cumulative incidence of cGVHD was 61% (n = 17) at a mean of 123 (range, 75-222) days after HSCT, including 41% (n = 7) patients with grades 2-3 (Table 2). Adiponectin levels were elevated in patients with established moderate to severe cGVHD (n = 17) compared to autologous controls (13.5 vs 7.6 µg/mL, *P* = 0.051, *t* test, Figure 3A, measured at disease maximum) but the difference was not significant. Adiponectin/BMI ratios were significantly higher in patients with established moderate/severe cGVHD than in autologous controls (0.59 vs 0.25, *P* = 0.006,, *t* test, Figure 3B, measured at disease maximum). No significant differences in resistin levels were observed compared to autologous controls or allogeneic patients with no cGVHD.

At the time point prior to the onset of cGVHD, future cGVHD patients had a significantly higher adiponectin/BMI ratio (0.9 vs 0.2, *P* = 0.032, *t* test) than autologous controls. Also, they had significantly higher adiponectin levels (16.9 vs 9.6 µg/mL, *P* = 0.042) and adiponectin/BMI ratios during aplasia (T+1) (0.8 vs 0.4, *P* = 0.038, *t* test).

Patients with cGVHD also had significantly higher FNC levels on admission (33.6 vs 7.8 µg/mL, *P* < 0.001, *t* test) and higher SAA levels 1 month after HSCT (13.8 vs 4.9, *P* = 0.033, *t* test) than autologous controls. When compared to non-cGVHD patients (n = 11), patients with cGVHD only had significantly reduced SAA at T0 and T+4 (9 vs 262, *P* = 0.034 and 5 vs 72, *P* = 0.010, *t* test).

### Patient outcome

**Figure 4:**
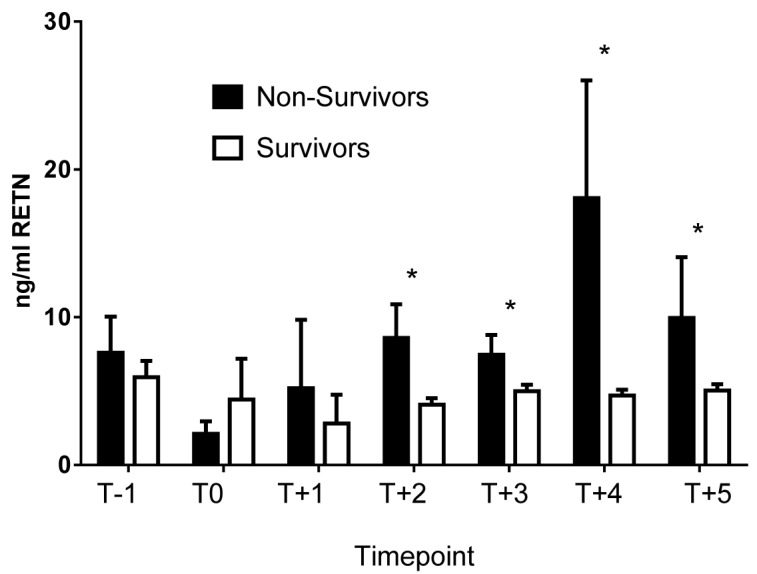
Resistin (RES) levels in relapsing non-survivors compared to surviving allogeneic patients.

Overall, 13 patients (32%) died within a mean of 8 months after HSCT. Prior to HSCT (T-1), only HPT levels were significantly higher in all patients subsequently dying than in all survivors of HSCT (135 vs 82 µg/mL, *P* = 0.013, *t* test). From T+2 through T+5, resistin, but not adiponectin or adiponectin/BMI ratio, was significantly higher in non-surviving allogeneic patients than in surviving allogeneic patients (Figure 4). Patient characteristics and clinical parameters that had an impact on overall survival in multivariate analyses were lower age at T0 (*P* = 0.052, multiple linear regression), but not sex, conditioning, HLA identity, stem cell source, or GVHD prophylaxis. Importantly, we did not observe differences in mortality, relapse, aGVHD, and cGVHD with regard to sex.

9 patients (22.5%) died within a mean of 4.7 months after HSCT due to relapse of their primary diseases. 4 patients (10%) died within a mean 14.5 months after HSCT due to causes that were not related to their primary disease (non-relapse mortality, NRM; causes of death: sarcoma, infection, cGVHD, aGVHD).

**Figure 5:**
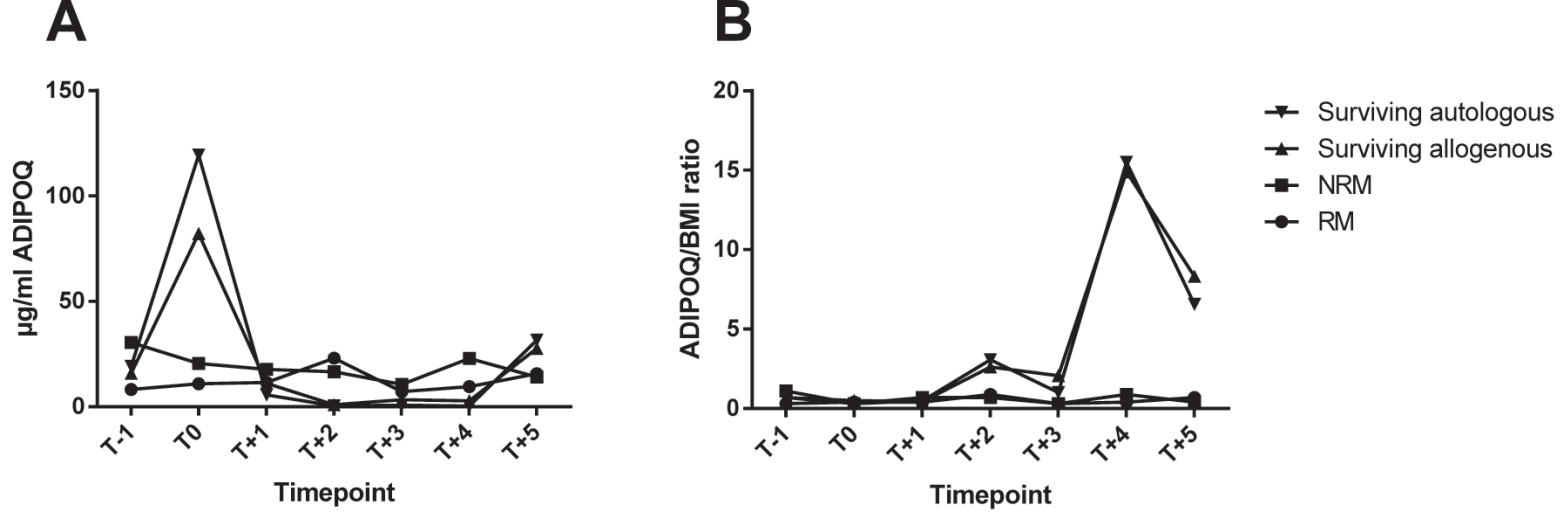
Adiponectin (ADIPOQ) (**A**) and ADIPOQ/body mass index (BMI) ratio (**B**) in surviving autologous, surviving allogenous, non-relapsing non-survivors (NRM), and relapsing non-survivors (RM) in the course of hematopoietic stem cell transplantation (HSCT).

Patients dying from relapse had significantly lower adiponectin levels (8.2 vs 30.4 µg/mL, *P* = 0.007) and adiponectin/BMI ratios (0.3 vs 1.1, *P* = 0.004) prior to HSCT (T-1) than patients experiencing NRM. Patients dying from relapse also had significantly lower adiponectin levels (8.2 µg/mL) and adiponectin/BMI ratio (0.3) at T-1 than surviving allogeneic (15.8 µg/mL, *P* = 0.030 and 0.7, *P* = 0.004, *t* test) and surviving autologous patients (19.2 µg/mL, *P* = 0.031 and 0.7, *P* = 0.021, *t* test). Throughout the whole study period, patients dying from relapse had higher adiponectin levels and adiponectin/BMI ratio than surviving allogeneic patients and all surviving patients (Figure 5). Allogeneic NRM patients (n = 4) did not show significantly different levels of adiponectin, resistin, or adiponectin/BMI ratio from surviving allogeneic patients. No impact of patient clinical characteristics on NRM or relapse was observed (multiple linear regression).

## Discussion

Our study was the first to show elevated adiponectin levels and adiponectin/BMI ratio in aGVHD and cGVHD and lower resistin levels in aGVHD. Also, patients dying from relapse had higher adiponectin levels and adiponectin/BMI ratio than surviving allogeneic patients. Adiponectin levels and adiponectin/BMI ratio were significantly higher in established moderate/severe cGVHD. These results are in accordance with the previous work by Nakasone (34). Furthermore, we reported a predictive value of adiponectin and resistin during aplasia (T+1) for subsequent development of aGVHD and cGVHD, suggesting that a change in adipokine levels precedes GVHD. This has not been shown before and might reflect pathophysiologic changes that take place at a very early stage before the appearance of clinical signs of GVHD. We interpret these results as a stronger, probably compensatory anti-inflammatory response in patients with a subsequent development of aGVHD directly after HSCT (at T+1), probably due to more tissue damage following conditioning and a consecutive pro-inflammatory up-regulation of resistin and SAA (at T+2 and T+3) at the initiation of aGVHD. Considering the anti-inflammatory properties of adiponectin and the pro-inflammatory properties of resistin, this phenomenon might represent two phases of the immunologic state activation of the white adipose tissue during GVHD.

An important finding of our study was that adiponectin levels and adiponectin/BMI ratios prior to HSCT (T-1) were significantly lower in patients subsequently dying from a relapse of their underlying disease. While this finding might not necessarily be causally connected to HSCT, it may still indicate that adipokines are involved in a broader regulation of the immune response during critical illnesses, with higher levels probably having a protective role. However, since we investigated HSCT patients only, this statement remains speculative. Resolution of aGVHD was accompanied by a significant reduction of adiponectin and resistin levels, as reported earlier (34).

The factors associated with improved overall survival were younger age and a normal BMI (18.5-25). While older age is unarguably associated with a worse outcome (1,3-5), conflicting data have been reported on BMI (46-48). Since most of our patients had normal body weight or were overweight (BMI 25-30) at admission, our findings cannot be applied to obese or underweight patients. Therefore, we could not confirm previously reported higher survival rates in patients with a BMI>30 (49).

We observed lower adiponectin levels and adiponectin/BMI ratios in patients who received myeloablative conditioning regimen compared to RIC. At present, there is no evidence if and how the conditioning regimen impacts adipocytes, the main source of adiponectin in humans. We can only speculate that myeloablative conditioning decreases adiponectin production by adipocytes or the adipocyte population itself. However, this observation might not indicate a causal relationship.

Annaloro et al (50) reported lower adiponectin levels in long-term allogeneic HSCT survivors with metabolic syndrome and higher BMI. Demographic characteristics of these patients were comparable to those of our participants, as were adiponectin levels. In this study, GVHD was not a predictor of metabolic syndrome in the subset of allogeneic HSCT recipients. Since adiponectin levels were inversely correlated with BMI but also were higher in GVHD patients with similar BMI, this finding might indicate that adiponectin is indeed involved in the pathophysiology of GVHD. However, Annaloro et al did not report any correlation with mortality or GVHD.

Adiponectin acts both pro- and anti-inflammatory, though most authors report only its anti-inflammatory properties. This makes the interpretation of the results particularly difficult: is adiponectin an additional factor causing GVHD or is it a key regulator inhibiting proinflammatory responses? Since in our study adiponectin/BMI ratios were higher in the non-relapsing and surviving patients, we propose that adiponectin has an anti-inflammatory effect during allogeneic HSCT. This assumption is supported by Katsiougiannis et al (51), who demonstrated that adiponectin protects salivary gland epithelial cells from apoptosis in Sjogren’s syndrome. Nakasone et al (34), on the other hand, proposed pro- and anti-inflammatory action in GVHD.

Proinflammatory action of adiponectin has been described mostly *in vitro* (52-54). Since recombinant adiponectin is very often contaminated by LPS (55) and its proinflammatory properties have not been described *in vivo*, we think that a relevant proinflammatory action of adiponectin in GVHD can currently be neglected.

The design of this study does not allow further in-depth investigation of the underlying mechanisms, so conclusions about them have to be drawn very carefully. The small sample size also poses a significant limitation, although the prospective design might counterbalance this. Furthermore, we speculated that adiponectin and resistin actions were not specific for GVHD, however we failed to provide evidence for this assumption. We cannot answer the very relevant question posed by Nakasone et al (34) whether high adiponectin levels in cGVHD are a primary or a secondary event. Another limitation is that we did not differentiate between different forms of adiponectin, since recent reports have shown that distinct isoforms might induce different effects *in vivo* and *in vitro* (56-58). We confirmed previous findings that adiponectin levels and adiponectin/BMI ratios were higher in women (37,38). Since in our study the autologous HSCT group consisted mainly of male patients, adiponectin levels in this group might be lower than in a group with equal sex distribution. This has to be taken into account in further studies. Although we did provide data on survival, the primary aim of the study was not to detect differences in mortality. However, some results indicate a possible involvement of adipokines in the pathomechanism of relapse, which might be a useful basis for further research.

Our data suggest a distinct expression of adiponectin and resistin during the course of HSCT. In this regard, further research is needed to clarify our observations. The next logical step would be to correlate the anti-inflammatory action in GVHD with specific adiponectin isoforms in a prospective study. In conclusion, adiponectin and resistin were altered during the occurrence of acute and chronic GVHD and were associated with overall survival and relapse mortality in patients undergoing allogeneic HSCT compared to autologous controls. Both cytokines could have a role in the pathophysiology of GVHD and should be evaluated in further studies.

## References

[1] Appelbaum FR (2007). Hematopoietic-cell transplantation at 50.. N Engl J Med.

[2] Passweg JR, Baldomero H, Gratwohl A, Bregni M, Cesaro S, Dreger P (2012). The EBMT activity survey: 1990-2010.. Bone Marrow Transplant.

[3] Lee SJ, Vogelsang G, Gilman A, Weisdorf DJ, Pavletic S, Antin JH (2002). A survey of diagnosis, management, and grading of chronic GVHD.. Biol Blood Marrow Transplant.

[4] Socie G, Stone JV, Wingard JR, Weisdorf D, Henslee-Downey PJ, Bredeson C (1999). Long-term survival and late deaths after allogeneic bone marrow transplantation. Late Effects Working Committee of the International Bone Marrow Transplant Registry.. N Engl J Med.

[5] Jagasia M, Arora M, Flowers ME, Chao NJ, McCarthy PL, Cutler CS (2012). Risk factors for acute GVHD and survival after hematopoietic cell transplantation.. Blood.

[6] Pasquini MC (2008). Impact of graft-versus-host disease on survival.. Best Pract Res Clin Haematol.

[7] Pidala J, Anasetti C, Jim H (2009). Quality of life after allogeneic hematopoietic cell transplantation.. Blood.

[8] Rouquette-Gally AM, Boyeldieu D, Gluckman E, Abuaf N, Combrisson A (1987). Autoimmunity in 28 patients after allogeneic bone marrow transplantation: comparison with Sjogren syndrome and scleroderma.. Br J Haematol.

[9] Cwynarski K, Goulding R, Pocock C, Dazzi F, Craddock C, Kaeda J (2001). Immune haemolytic anaemia following T cell-depleted allogeneic bone marrow transplantation for chronic myeloid leukaemia: association with leukaemic relapse and treatment with donor lymphocyte infusions.. Bone Marrow Transplant.

[10] Rouquette-Gally AM, Boyeldieu D, Prost AC, Gluckman E (1988). Autoimmunity after allogeneic bone marrow transplantation. A study of 53 long-term-surviving patients.. Transplantation.

[11] Filipovich AH, Weisdorf D, Pavletic S, Socie G, Wingard JR, Lee SJ (2005). National Institutes of Health consensus development project on criteria for clinical trials in chronic graft-versus-host disease: I. Diagnosis and staging working group report.. Biol Blood Marrow Transplant.

[12] Jagasia MH Greinix HT Arora M Williams KM Wolff D Cowen EW National Institutes of Health Consensus Development Project on Criteria for Clinical Trials in Chronic Graft-versus-Host Disease: I. The 2014 Diagnosis and Staging Working Group report Biol Blood Marrow Transplant 2015 21(3)389 401 e381 10.1016/j.bbmt.2014.12.001 25529383 PMC4329079

[13] Goker H, Haznedaroglu IC, Chao NJ (2001). Acute graft-vs-host disease: pathobiology and management.. Exp Hematol.

[14] Liu W, Xiao X, Demirci G, Madsen J, Li XC (2012). Innate NK cells and macrophages recognize and reject allogeneic nonself in vivo via different mechanisms.. J Immunol.

[15] Fantuzzi G (2008). Adiponectin and inflammation: consensus and controversy.. J Allergy Clin Immunol.

[16] Zacharioudaki V, Androulidaki A, Arranz A, Vrentzos G, Margioris AN, Tsatsanis C (2009). Adiponectin promotes endotoxin tolerance in macrophages by inducing IRAK-M expression.. J Immunol.

[17] Behnes M, Brueckmann M, Lang S, Putensen C, Saur J, Borggrefe M (2012). Alterations of adiponectin in the course of inflammation and severe sepsis.. Shock.

[18] Trellakis S, Rydleuskaya A, Fischer C, Canbay A, Tagay S, Scherag A (2012). Low adiponectin, high levels of apoptosis and increased peripheral blood neutrophil activity in healthy obese subjects.. Obes Facts..

[19] Robinson K, Prins J, Venkatesh B (2011). Clinical review: adiponectin biology and its role in inflammation and critical illness.. Crit Care.

[20] Yokota T, Oritani K, Takahashi I, Ishikawa J, Matsuyama A, Ouchi N (2000). Adiponectin, a new member of the family of soluble defense collagens, negatively regulates the growth of myelomonocytic progenitors and the functions of macrophages.. Blood.

[21] Wang Y, Lam KS, Xu JY, Lu G, Xu LY, Cooper GJ (2005). Adiponectin inhibits cell proliferation by interacting with several growth factors in an oligomerization-dependent manner.. J Biol Chem.

[22] Mandal P, Pratt BT, Barnes M, McMullen MR, Nagy LE (2011). Molecular mechanism for adiponectin-dependent M2 macrophage polarization: link between the metabolic and innate immune activity of full-length adiponectin.. J Biol Chem.

[23] Park PH, Huang H, McMullen MR, Mandal P, Sun L, Nagy LE (2008). Suppression of lipopolysaccharide-stimulated tumor necrosis factor-alpha production by adiponectin is mediated by transcriptional and post-transcriptional mechanisms.. J Biol Chem.

[24] Nishiwaki S, Terakura S, Ito M, Goto T, Seto A, Watanabe K (2009). Impact of macrophage infiltration of skin lesions on survival after allogeneic stem cell transplantation: a clue to refractory graft-versus-host disease.. Blood.

[25] Haniffa M, Ginhoux F, Wang XN, Bigley V, Abel M, Dimmick I (2009). Differential rates of replacement of human dermal dendritic cells and macrophages during hematopoietic stem cell transplantation.. J Exp Med.

[26] Okamoto Y, Folco EJ, Minami M, Wara AK, Feinberg MW, Sukhova GK (2008). Adiponectin inhibits the production of CXC receptor 3 chemokine ligands in macrophages and reduces T-lymphocyte recruitment in atherogenesis.. Circ Res.

[27] Okamoto Y, Christen T, Shimizu K, Asano K, Kihara S, Mitchell RN (2009). Adiponectin inhibits allograft rejection in murine cardiac transplantation.. Transplantation.

[28] Stofkova A (2010). Resistin and visfatin: regulators of insulin sensitivity, inflammation and immunity.. Endocr Regul.

[29] Haluzik M, Haluzikova D (2006). The role of resistin in obesity-induced insulin resistance.. Curr Opin Investig Drugs.

[30] Jamaluddin MS, Weakley SM, Yao Q, Chen C (2012). Resistin: functional roles and therapeutic considerations for cardiovascular disease.. Br J Pharmacol.

[31] Tanaka N, Kusunoki N, Kusunoki Y, Hasunuma T, Kawai S (2013). Resistin is associated with the inflammation process in patients with systemic autoimmune diseases undergoing glucocorticoid therapy: comparison with leptin and adiponectin.. Mod Rheumatol.

[32] Rae C, Graham A (2006). Human resistin promotes macrophage lipid accumulation.. Diabetologia.

[33] Qatanani M, Szwergold NR, Greaves DR, Ahima RS, Lazar MA (2009). Macrophage-derived human resistin exacerbates adipose tissue inflammation and insulin resistance in mice.. J Clin Invest.

[34] Nakasone H, Binh PN, Yamazaki R, Tanaka Y, Sakamoto K, Ashizawa M (2011). Association between serum high-molecular-weight adiponectin level and the severity of chronic graft-versus-host disease in allogeneic stem cell transplantation recipients.. Blood.

[35] Hirose H, Yamamoto Y, Seino-Yoshihara Y, Kawabe H, Saito I (2010). Serum high-molecular-weight adiponectin as a marker for the evaluation and care of subjects with metabolic syndrome and related disorders.. J Atheroscler Thromb.

[36] Arita Y, Kihara S, Ouchi N, Takahashi M, Maeda K, Miyagawa J (1999). Paradoxical decrease of an adipose-specific protein, adiponectin, in obesity.. Biochem Biophys Res Commun.

[37] Cnop M, Havel PJ, Utzschneider KM, Carr DB, Sinha MK, Boyko EJ (2003). Relationship of adiponectin to body fat distribution, insulin sensitivity and plasma lipoproteins: evidence for independent roles of age and sex.. Diabetologia.

[38] Staiger H, Tschritter O, Machann J, Thamer C, Fritsche A, Maerker E (2003). Relationship of serum adiponectin and leptin concentrations with body fat distribution in humans.. Obes Res.

[39] Przepiorka D, Weisdorf D, Martin P, Klingemann HG, Beatty P, Hows J (1995). 1994 Consensus Conference on Acute GVHD Grading.. Bone Marrow Transplant.

[40] Glucksberg H, Storb R, Fefer A, Buckner CD, Neiman PE, Clift RA (1974). Clinical manifestations of graft-versus-host disease in human recipients of marrow from HL-A-matched sibling donors.. Transplantation.

[41] Tomblyn M, Chiller T, Einsele H, Gress R, Sepkowitz K, Storek J (2009). Guidelines for preventing infectious complications among hematopoietic cell transplantation recipients: a global perspective.. Biol Blood Marrow Transplant.

[42] Fantuzzi G (2013). Adiponectin in inflammatory and immune-mediated diseases.. Cytokine.

[43] Yamauchi T, Kamon J, Waki H, Terauchi Y, Kubota N, Hara K (2001). The fat-derived hormone adiponectin reverses insulin resistance associated with both lipoatrophy and obesity.. Nat Med.

[44] Simpson NS, Banks S, Arroyo S, Dinges DF (2010). Effects of sleep restriction on adiponectin levels in healthy men and women.. Physiol Behav.

[45] Silha JV, Krsek M, Skrha JV, Sucharda P, Nyomba BL, Murphy LJ (2003). Plasma resistin, adiponectin and leptin levels in lean and obese subjects: correlations with insulin resistance.. Eur J Endocrinol.

[46] Nikolousis E, Nagra S, Paneesha S, Delgado J, Holder K, Bratby L (2010). Allogeneic transplant outcomes are not affected by body mass index (BMI) in patients with haematological malignancies.. Ann Hematol.

[47] Navarro WH, Loberiza FR, Bajorunaite R, van Besien K, Vose JM, Lazarus HM (2006). Effect of body mass index on mortality of patients with lymphoma undergoing autologous hematopoietic cell transplantation.. Biol Blood Marrow Transplant.

[48] Lange BJ, Gerbing RB, Feusner J, Skolnik J, Sacks N, Smith FO (2005). Mortality in overweight and underweight children with acute myeloid leukemia.. JAMA.

[49] Jaime-Perez JC, Colunga-Pedraza PR, Gutierrez-Gurrola B, Brito-Ramirez AS, Gutierrez-Aguirre H, Cantu-Rodriguez OG (2013). Obesity is associated with higher overall survival in patients undergoing an outpatient reduced-intensity conditioning hematopoietic stem cell transplant.. Blood Cells Mol Dis.

[50] Annaloro C, Usardi P, Airaghi L, Giunta V, Forti S, Orsatti A (2008). Prevalence of metabolic syndrome in long-term survivors of hematopoietic stem cell transplantation.. Bone Marrow Transplant.

[51] Katsiougiannis S, Tenta R, Skopouli FN (2010). Activation of AMP-activated protein kinase by adiponectin rescues salivary gland epithelial cells from spontaneous and interferon-gamma-induced apoptosis.. Arthritis Rheum.

[52] Tomizawa A, Hattori Y, Kasai K, Nakano Y (2008). Adiponectin induces NF-kappaB activation that leads to suppression of cytokine-induced NF-kappaB activation in vascular endothelial cells: globular adiponectin vs. high molecular weight adiponectin.. Diab Vasc Dis Res.

[53] Haugen F, Drevon CA (2007). Activation of nuclear factor-kappaB by high molecular weight and globular adiponectin.. Endocrinology.

[54] Neumeier M, Weigert J, Schaffler A, Wehrwein G, Muller-Ladner U, Scholmerich J (2006). Wet al. Different effects of adiponectin isoforms in human monocytic cells.. J Leukoc Biol.

[55] Turner JJ, Smolinska MJ, Sacre SM, Foxwell BM (2009). Induction of TLR tolerance in human macrophages by adiponectin: does LPS play a role?. Scand J Immunol.

[56] Wedellova Z, Kovacova Z, Tencerova M, Vedral T, Rossmeislova L, Siklova-Vitkova M (2013). The impact of full-length, trimeric and globular adiponectin on lipolysis in subcutaneous and visceral adipocytes of obese and non-obese women.. PLoS ONE.

[57] Chedid P, Hurtado-Nedelec M, Marion-Gaber B, Bournier O, Hayem G, Gougerot-Pocidalo MA (2012). Adiponectin and its globular fragment differentially modulate the oxidative burst of primary human phagocytes.. Am J Pathol.

[58] Brochu-Gaudreau K, Rehfeldt C, Blouin R, Bordignon V, Murphy BD, Palin MF (2010). Adiponectin action from head to toe.. Endocrine.

